# Hiding in Plain Sight: Catastrophic Diethylene Glycol Poisoning in Children

**DOI:** 10.34067/KID.0000000000000269

**Published:** 2023-10-13

**Authors:** Mark A. Perazella

**Affiliations:** Section of Nephrology, Yale School of Medicine, New Haven, Connecticut

**Keywords:** diethylene glycol poisoning, AKI, medications, adverse drug events, neurotoxicity

Medications are one of the most important therapies available to prevent and treat a wide variety of health conditions. The important characteristics of pharmaceutical agents are that they should be both effective and safe. Without these qualities, significant morbidity and mortality would result when patients who have potentially treatable diseases are prescribed substandard drugs. Thus, a strong and well-supported regulatory system must be in place to ensure that medications meet these two critical standards. In the United States, the Food and Drug Administration (FDA) assumes this regulatory responsibility. Despite various criticisms about the speed and efficiency of FDA's drug approval process, it plays a critical role in ensuring that medications are both effective and safe. Unfortunately, regulatory system oversight across the globe is very inconsistent and thus may be substandard or even deliberately fraudulent, risking potentially severe drug complications. The resulting complications range from ineffective treatment of various diseases, development of drug resistance, and catastrophic poisoning from dangerous intoxicants. Pharmaceutical fraud, due to either human error or deliberate substitution of unsafe substances, is particularly concerning in countries with weak or incompetent regulatory systems that do not provide adequate quality checks, ultimately allowing poisoning outbreaks.^1^

While medications may contain fraudulent ingredients that lack efficacy, this editorial will focus on poisonings that develop from harmful contaminants, such as diethylene glycol (DEG). Sadly, many patients with severe poisoning from DEG have been described. Recently, a cluster of cases (*n*=78) of AKI among young children due to poisoning from DEG that contaminated children's syrup-based medications was reported in Gambia, a West African country.^2^ A fatality rate of >80% was associated with anuric AKI with the use of these DEG-contaminated medications, which were ultimately tracked to a single pharmaceutical manufacturer (Maiden Pharmaceuticals Limited, Haryana, India). In Nigeria, AKI and death due to teething syrup containing DEG was described in 84 children who were exposed to this contaminant.^3,4^ Eventually, inspectors traced the DEG poisoning to pharmaceutical fraud committed by a chemical dealer in Lagos that intentionally substituted DEG for another solvent.^3,4^ Another heartbreaking DEG poisoning occurred in Panama. A total of 219 deaths occurred from AKI due to DEG that was present in cough syrup when a Chinese chemical manufacturer sold DEG substituted as pharmaceutical-grade glycerin to a European company.^5,6^ DEG was illicitly used as a solvent in the cough syrup. Because it is estimated that >60,000 bottles of cough syrup and other lotions were contaminated with DEG, the Ministry of Health and the World Health Organization suggest that the 219 deaths likely reflect only a fraction of the total mortality due to this poisoning.^6^

In this issue of *Kidney360*, Murtalibova and colleagues report a heartbreaking epidemic of AKI, anion gap metabolic acidosis, neurological dysfunction, and death due to DEG poisoning—this time in children in Uzbekistan and adjoining areas of Tajikistan who were exposed to a brand of contaminated antipyretic and cough suppressant medication.^7^ Their report describes 50 children from two medical facilities in Tashkent, Uzbekistan. Most of the children required mechanical ventilation and dialysis, and 36% of the children ultimately died. Kidney function ultimately recovered in 58% of the children. This publication importantly shines a light on the lack of adequate oversight and quality control of drugs by regulatory agencies and the devastation that ultimately results.

The adverse clinical manifestations of DEG result from the formation of the metabolite diglycolic acid (DGA).^8^ This toxic metabolite is speculated to cause the end-organ damage observed with DEG poisoning.^8^ The most prominent clinical manifestation of DEG poisoning is nephrotoxicity (AKI and anion gap metabolic acidosis), which follows gastrointestinal symptoms, including nausea/vomiting, abdominal pain, and diarrhea. DGA is a toxin that causes mitochondrial injury after uptake and concentration within renal proximal tubular epithelial cells. Severe acute tubular injury and coagulative cortical necrosis have also been reported in the setting of DEG intoxication.^8,9^ Subsequently, progressive neurological dysfunction follows and manifests as encephalopathy, multiple peripheral and cranial neuropathies, quadriparesis, and other neuropathic findings. Unfortunately, despite supportive measures, including hemo- and peritoneal dialysis and inhibition of alcohol dehydrogenase, which metabolizes DEG to DGA, the mortality rate is high for DEG poisoning.

Prevention of DEG and other poisonings requires a significant international effort to promote consistent adherence to medication-manufacturing best practices and regulatory enforcement of the pharmaceutical industry and drug manufacturers. Because DEG is used as a solvent/excipient in place of propylene glycol and glycerin, attentive surveillance of products that use these substances would be welcomed. Furthermore, drug manufacturers should be mandated to strictly follow quality assurance guidance (Current Good Manufacturing Practice) set forth by regulatory authorities, such as the FDA and the World Health Organization.^10^ Appropriate monitoring and testing of the products synthesized by these drug manufacturers, including supply chain distributors, should be a standard part of the quality control surveillance process. Obviously, part of this regulatory process should focus on identifying contaminated products and targeting the culprit manufacturers and/or suppliers. An equally important part of this process is ensuring that a vigorous adverse drug events reporting system is widely available to detect patients early before epidemic-like adverse events occur. Ultimately, the goal is to prevent or at least reduce the occurrence of these poisonings.
